# Vitamin D Role in Childhood Mite Allergy and Allergen Immunotherapy (AIT)

**DOI:** 10.3390/biomedicines11061700

**Published:** 2023-06-13

**Authors:** Claudia Petrarca, Davide Viola

**Affiliations:** Department of Medicine and Science of Aging, G. d’Annunzio University, 66100 Chieti, Italy

**Keywords:** vitamin D, mite allergy, *Dermatophagoides*, monomeric allergoid, immunotherapy, rhinitis, antihistamine, immunoglobulin, IgE, asthma, corticosteroid, children

## Abstract

The post hoc analysis presented here aimed to address the influence of endogenous vitamin D in the immunological mechanism underlying effective mite allergoid immunotherapy (AIT). Previously, we have shown that in allergic children, after 12 months of this immunoactive treatment, functionally potentiated memory regulatory T cells are identified. Indeed, AIT is the only known treatment that is able to reshape the detrimental immune response against the allergen into a non-noxious one. Besides, VD is widely considered an immunoregulatory molecule that is endogenously produced and exogenously provided by foods and supplements that might interact with the AIT mechanism, thus affecting its outcome. Therefore, a post hoc analysis of the clinical and immunological data from three different cohorts of allergic patients was performed. One cohort (N = 70) was on a standard symptom-controlling pharmacological treatment, while the other two (N = 60 and N = 35) were treated with AIT for 12 months. In the first cohort, a lower mean endogenous VD level (<22 ng/mL) was observed along with worse symptoms and a greater use of medications. Remarkably, the comparison between two sub-cohorts of patients with a serum VD level above (N = 32) or below (N = 28) a cut-off value set at the mean value (27 ng/mL) revealed that optimal improvement of all clinical and immune parameters was achieved (as expected from effective AIT), irrespective of the VD level. Notably, the third analysis, carried out on one cohort of AIT patients that were also concomitantly taking VD3 as a food supplement (N = 19), was distinguished by an uppermost overall treatment outcome (the amelioration of symptoms, the lowest medication requirements, and a reduction in the total and allergen-specific IgE levels), as well as an increase in the allergen-specific tolerogenic memory T regulatory cells. These findings suggest that the endogenous VD level affects the allergy severity and allergen immunotherapy effectiveness. In addition, VD3 might be investigated as an add-on supplement to obtain the best out of immunotherapy in VD-deficient/-insufficient allergic patients. The immunogenic, but low-allergenic, mite allergoid used as the bioactive agent might have contributed to minimizing the allergic response and highlighting the immunological effects described here.

## 1. Introduction

Vitamin D3 (1,25-dihydroxy vitamin D, VD3), the active form of vitamin D, is a compelling immunoregulatory molecule [1]. Immune cells are sensitive to VD3 after it binds to the cytoplasmic vitamin D receptor (VDR), which allows it to enter the nucleus, where it forms a new complex with the retinoic acid receptor (RXR). In this way, VD3-VDR-RXR binds to VD response elements (VDRE) located in the promoter of genes playing immune stimulatory or inhibitory roles. VDR is expressed constitutively by antigen-presenting cells (APCs) such as macrophages and dendritic cells (DCs), and in an inducible way by activated T regulatory cells (Tregs). Monocytes, macrophages, and T cells can occasionally express enzymes (CYP27A1 and/or CYP27B1) that catalyze the conversion of the inactive precursor into active VD3. Immune-cell-produced VD3 acts locally in an autocrine or paracrine way to regulate the immune response, and it exerts pleiotropic effects on both innate and acquired pathways of immunity [2,3]. In macrophages and monocytes, VD3 positively regulates its own effects by increasing the expression of VDR and activating enzymes; it also induces monocyte proliferation and the expression of pro-inflammatory interleukin (IL)-1 by macrophages. VD3 hinders the maturation of DC, causing a switch in the cytokine production pattern and resulting in lower amounts of pro-inflammatory IL-12 and higher levels of inhibitory/regulatory IL-10. An additional notable effect of VD3 on innate cells is a reduction in IL-4 release [4], a typical cytokine involved in allergic responses. Furthermore, VD3 attenuates the proliferation of CD4+ (helper) and the detrimental activity of CD8+ (cytotoxic) effector T cells, influencing the decreased production of the cytokine IL-2, which plays a role in the pan T cell growth factor, and the pro-inflammatory interferon γ (IFN-γ), the Th1 cell differentiation factor. Furthermore, VD3 blocks B cell proliferation, differentiation, and immunoglobulin production [4] and indirectly blocks the activity of Th2 cells, effector allergen-specific cells involved in allergic responses. Most importantly, VD3 promotes the development of innate and IL-10-producing inducible Tregs, which play pivotal roles in allergy development and control [5]. The induction of a durable tolerance towards an allergen is the main scope of allergen immunotherapy (AIT), which is the only treatment shown to have this capacity. The mechanistic interplay is accepted to rely on the elicitation of several subtypes of central and peripheral allergen-specific Tregs that, consequently, increase the production of the inhibitory cytokine IL-10 and transforming growth factor β (TGF-β). Lastly, AIT can suppress aberrant Th2 responses and induce an isotype switch of allergen-specific IgE to IgG production by plasma cells [6,7].

The clinical efficacy of AIT has been proven. For illustration, during grass pollen AIT, the number of Th2-type memory cells decreases in conjunction with an increase in Treg and Th1 cells [8], and the Treg results were increased in the nasal mucosa of treated patients [9]. Recently, we showed that a 1-year treatment with mite AIT was effective at promoting an increase in functional memory Tregs, which are characterized by a greater number of surface inhibitory functional markers [10].

It is known that, even if AIT results in the clear attenuation or disappearance of symptoms, up to five years of treatment and a tight adherence are required, and non-responsive patients occur at a 30% frequency [11].

Hence, it would be important to find a naturally occurring add-on compound that is able to boost the immunomodulatory activity of AIT and favor its therapeutic effects for a larger number of patients at an earlier time. Vitamin D3 might indeed play such a role.

However, there are conflicting findings on its potential role in the risk, prevention, and amelioration of allergic diseases, which likely reflect the patients’ age and atopy status, the site of manifestation, and the geographic area (Table 1).

Contradictory associations have been described among the data referring to the VD3 status of the mother. A higher intake of VD-rich foods during pregnancy had a preventive effect against allergies in the mother’s own atopic children [12]. However, VD3 supplementation appeared to inconveniently promote childhood atopic dermatitis [13]. Another study showed that VD3 supplementation reduced the risk of the exacerbation of asthma, atopic dermatitis, and rhinitis symptoms in children, and endogenous levels of VD appeared to be a critical factor in allergy onset, too [14]. More recently, the findings of a clinical trial did not support the use of vitamin D3 supplementation to prevent severe asthma exacerbations in a group of patients with low vitamin D levels [15].

Remarkably, in asthmatic children undergoing AIT, a combination treatment with VD3 further enhanced the increase in Tregs, IL-10, and TGF-β [18,19] and reduced nasal symptoms and asthma [16]. Conversely, in non-atopic toddlers, VD3 supplementation was associated with a higher risk of developing asthma [17], allergic rhinitis, and atopic dermatitis.

Evidently, the role of endogenous VD in allergy severity and AIT has not been clearly assessed so far. Previously, our group described an adjuvant role for VD3 supplementation in a murine model of type I mite allergies when administered in combination with Der p 1 immunotherapy [20]. Starting from these premises, three data sets regarding three cohorts of allergic children (eligible or not for AIT) were retrospectively analyzed to assess whether endogenous VD or supplemented VD3 might contribute to ameliorating the clinical outcome and/or promoting the functionality of regulatory T cells in this unexplored setting.

## 2. Patients, Materials, and Methods

### 2.1. Inclusion Criteria

The inclusion criteria included patients with IgE sensitization and symptoms of allergic rhinitis, with or without concomitant well-controlled asthma symptoms, who were considered eligible for AIT. All participants were enrolled voluntarily based upon their written informed consent, as well as that of their parents/tutors at the Pediatric Allergology and Respiratory Unit, University Hospital of Chieti (Italy).

### 2.2. Diagnosis

Mite allergies and sensitization to *D. farinae* (Der f) and *D. pteronyssinus* (Der p) were assessed by a positive skin-prick test and a serum-specific IgE level ≥ 0.7 kUA/L. An allergy to inhalants and food allergens was measured using the ImmunoCAP system (Phadia, Thermo-Scientific, Milan, Italy), which detects specific IgE values in the range between 0 and 100 kUA/L. The diagnosis and severity of the rhinitis were determined according to the ARIA classification [21] by applying a 5-point scoring system: 0, no rhinitis; 1, intermittent and mild rhinitis; 2, intermittent and moderate/severe rhinitis; 3, persistent and mild rhinitis; and 4, persistent and moderate/severe rhinitis. The diagnosis and severity of the asthma were assessed according to the GINA guidelines [22]: based on the medical history, physical examination findings, global spirometry, a post-bronchodilator FEV 1 ≥ 70% of the predicted volume, asthma control test (ACT), and inhaled corticosteroids/long-acting β2 agonist (ICS/LABA) and anti-leukotriene consumption. A visual analogue scale (VAS) was used for the past month’s severity rating by using a 10 cm scale, where 0 cm means no symptoms and 10 cm corresponds to the highest level of symptoms. A medication score was assigned by counting the number of patients using antihistamines and local or systemic steroids.

### 2.3. Exclusion Criteria 

The exclusion criteria included a reduced lung function (FEV1 < 70% of the predicted value), a history of uncontrolled severe asthma (within 3 months before screening), an HDM-SIT during the last 5 years, major conditions of the oral cavity, systemic diseases, the usage of medications that are able to interfere with treatment, and the occurrence of systemic side effects after the administration of each dose of AIT.

### 2.4. AIT Treatment Protocol

Sublingual immunotherapy (SLIT) was performed with the monomeric allergoid LAIS^®^ (Lofarma SpA, Milan, Italy), containing 50% each of Der f and Der p house dust mites. Each day, 1 tablet was kept under the tongue for 1–3 min and then swallowed. The patients were asked to refrain from drinking or eating for 15 min. The SLIT regimen consisted of an induction phase (500 AU/day) followed by a maintenance phase. Then, all children assumed the maintenance dose of 4000 AU/week for at least 12 months, as already described [10].

### 2.5. VD3 Supplementation

A VD3 food supplement for the prevention of pediatric musculoskeletal disorders was used.

### 2.6. Serum VD Level

VD levels were determined using an ELISA test (LIAISON^®^ 25-OH Vit Assay Kit; DiaSorin S.p.A., Saluggia VC, Italy), with a detection range between 7.0 and 150 ng/mL.

### 2.7. Memory T Regulatory Cell Flow Cytometry Assessment

T regulatory cells were analyzed to evaluate the peripheral effector memory Tregs expressing HLA-DR [10] as a surrogate endpoint. Briefly, peripheral blood mononuclear cells (PBMC) were stained using a panel of lyophilized reagents (Lyotube #624637, BD Biosciences, Milan, Italy) for 30 min at 4 °C in the dark. Then, the samples underwent an erythrocyte-lyse step with 1X lysing solution for 15 min at RT. Next, the samples were centrifuged and washed. For each sample, 1.5 × 10^5^ events were acquired by the flow cytometry instrument FACSCanto. The threshold was placed on the forward scatter channel (FSC). To ensure the correct identification of negative and positive populations, cells were plotted using a dot-plot bi-exponential display. Instrument performances, data reproducibility, and fluorescence calibrations were sustained and checked by the cytometer setup and tracking module and further validated by the acquisition of 8-peak Rainbow Beads (Spherotech, BD Biosciences, Milan, Italy). Non-specific fluorescence was achieved using the fluorescence-minus-one (FMO) control method. The compensation was assessed using CompBeads and single-stained fluorescent samples. The data were analyzed using FlowJo v. 8.8.6 (TreeStar, Ashland, OR, USA). Reagents, instruments, and software were purchased from BD Biosciences (Milan, Italy), if not otherwise indicated. The HLA-DR surface expression, evaluated in terms of the mean fluorescence intensity (MFI), was normalized based on the relative expression of the respective CD4^neg^ lymphocyte compartment. The Treg data are expressed as the median ± interquartile range (IQR).

## 3. Methodology of Post Hoc Analysis

**Figure 1:**
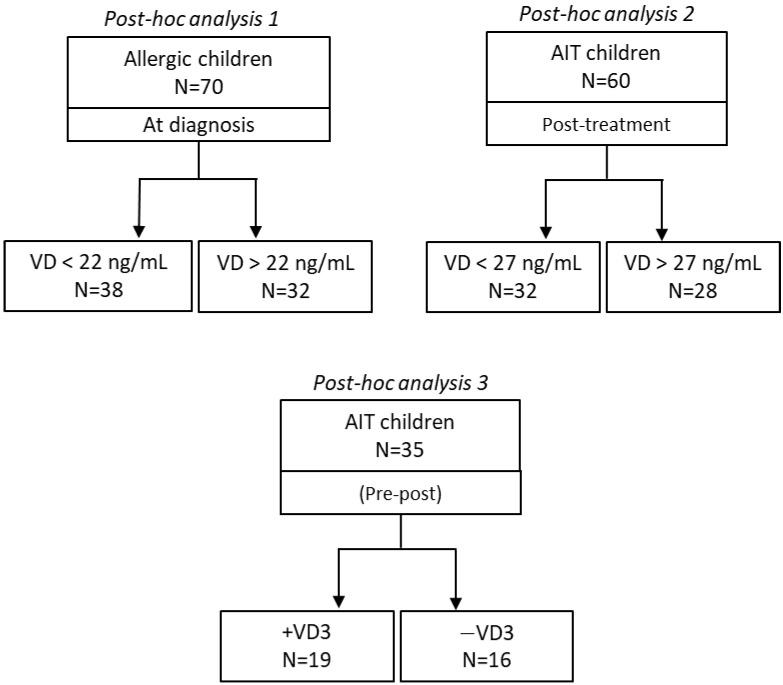
Outlines of the three post hoc analyses.

The patient population was selected from the database of the Pediatric Allergology and Respiratory Unit, University of Chieti (Italy), which contained all consecutive subjects with symptoms of allergies who contacted the diagnostic unit from September 2019 to June 2021. The specific diagnostic procedures and methods were protocolized as briefly reported in Section 2 and as described in a previous study [10]. One hundred and sixty-five consecutive patients, diagnosed with house dust mite (HDM) allergies, underwent symptomatic therapy or AIT. Three different cohorts were selected post hoc to compare the dichotomous parameter of interest (Figure 1). The first cohort was placed on a standard symptom-controlling pharmacological treatment (N = 70). The second and third cohorts (N = 60 and N = 35, respectively) were treated with AIT for 12 months. The latter cohort (N = 35) was built up to generate two independent sub-cohorts that were comparable in terms of anthropometric parameters and medical history (Table 2).

The relevant anthropometric and clinical data, as well as the laboratory and respiratory data, of the patients involved in this analysis are shown in Table 2.

The clinical and laboratory data were expressed as means ± standard deviations (SD), unless otherwise indicated. All the measured parameters showed a non-parametric distribution, according to Shapiro–Wilk’s criteria. A statistical comparative analysis between two datasets, below and above the VD cut-off value or ±VD3 food supplement, was performed using the Wilcoxon signed-rank test. Statistical analyses were performed using the program Statistical Package for Social Science (SPSS, Chicago, IL, USA). *p* Values < 0.05 were considered statistically significant.

## 4. Results

### 4.1. Endogenous VD in Allergic Children—Post Hoc Analysis 1

Allergic patients in the present study (N = 70) were found to be characterized by heterogeneous endogenous levels of serum VD ranging from 17 to 27 ng/mL, hence covering biologically deficient, insufficient, and sufficient values [23] (Table 3).

By setting a cut-off value for the serum VD concentration equal to the observed mean value (22 ng/mL), an analysis of categorical variables revealed that the group characterized by the lower serum VD (N = 38) showed a significantly higher level of IgE (total and specific) and need for ICS-LABA, as well as a higher VAS score. Reversely, the group with a high VD (N = 32) showed sufficient endogenous VD levels and the lowest IgE levels and medication demand (Table 4). The two sub-cohorts were otherwise comparable in terms of anthropometric parameters and medical history (Table 2).

### 4.2. Endogenous VD in AIT Children—Post Hoc Analysis 2

The serum VD level, allergy, and respiratory data of the AIT group of patients (N = 60) that were used for the diagnosis and the AIT prescription, and the data after 12 months of treatment, are shown in Table 5.

Notably, a wide range of serum VD levels was also detected in this cohort of patients at baseline and after 12 months post-effective AIT treatment (Table 5).

Remarkably, a comparison between these two endogenous VD sub-cohorts, using the mean value of 27 ng/mL as the cut-off, showed no significant differences in the overall AIT clinical outcome based on an improvement in the respiratory clinical scores (ARIA, ACT), except for oral antihistamines and VAS. In addition, the allergen IgE serum level was not significantly different when comparing the two sub-cohorts (Table 6).

### 4.3. Exogenous Supplementation of VD3 in AIT Children—Post Hoc Analysis 3

**Figure 2:**
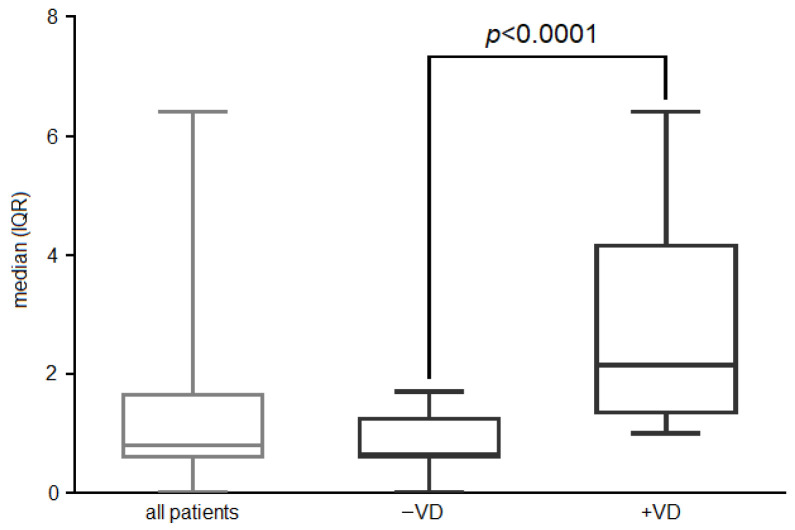
HLA expression level expressed as median (IQR) of effector memory Tregs in AIT-treated patients compared to VD3-supplemented patients (or those with an endogenous VD > 30 ng/mL).

After 12 months of treatment, the group of AIT patients supplemented with VD3 (+VD3, N = 19) showed a significant increase in their serum VD levels (baseline: 25.2 ± 2.4 vs. post-treatment: 36.1 ± 2.8 ng/mL) (*p* < 0.0001). Instead, no significant changes were found between the same time points in the other sub-cohort of non-supplemented AIT children. All relevant clinical parameters (ARIA, antihistamines, nasal corticosteroids, and ICS-LABA consumption) were improved in both groups of patients, regardless of VD3, as expected for an effective AIT outcome (Table 7). Alongside this, peripheral effector memory Tregs were also induced in both sub-cohorts of patients (all patients), confirming a previous finding of our group regarding the tolerogenic role of AIT (Figure 2).

Interestingly, VD3 supplementation was associated with a significant increase in the endogenous VD level up to fully sufficient values (>30 ng/mL) (Table 7). In this sub-cohort of patients, the uppermost amelioration of allergic symptoms, the lowest corticosteroid requirement, and the steepest diminution in total and specific anti-Df IgE levels, which is the global AIT outcome, were observed after 12 months of treatment. In fact, the clinical improvement according to ARIA and the reduction in antihistamine consumption were significantly greater in the sub-cohort group that took the VD3 food supplement as compared to the group that did not (*p* < 0.05). A reduced ICS-LABA requirement was registered for the +VD3 sub-cohort group compared to the other one (*p* < 0.0001) (Table 8).

Furthermore, a significant diminution in the serum levels for the total IgE and specific anti-Df IgE levels was observed only in the +VD3 cohort group after AIT (Table 9).

In addition, endogenous VD appeared to be involved in the extra increase in the functionality of peripheral effector memory Tregs (HLA-DR expression level on CD4^+^CD25^+^CD127^neg^CD39^+^CD45RA^neg^HLA-DR^+^ peripheral blood lymphocytes) in sub-cohort +VD3 (endogenous level: VD > 30 ng/mL), compared to those without supplementation (*p* < 0.0001) (Figure 2).

A schematization of the overall results of the three data sets analyzed is shown in Table 10.

## 5. Discussion

The rationale of this post hoc analysis was to address the influence of endogenous vitamin D in children with a respiratory mite allergy that are being treated with allergen immunotherapy (AIT). Indeed, VD has been described as a pleiotropic immunoregulator. In fact, other investigators have shown that it promotes the maturation of Tregs by inducing the surface expression and/or secretion of inhibitory cytokines, which play a pivotal role in allergy control. Furthermore, VD exerts an indirect blocking activity on Th2 effector cell and B cell proliferation, differentiation, and IgE production [3]. Despite these advancements in the comprehension of immunological mechanisms, the role of VD in human allergic sensitization and symptomatic diseases is difficult to disentangle. In fact, variable and incoherent findings are described in clinical settings and trials. Contradictory associations have been described between different VD levels during pregnancy. A higher intake of VD-rich foods during pregnancy has a preventive effect, as it reduces the incidence of asthma, recurrent dyspnea, allergic rhinitis, and food allergies. However, high VD3 supplementation appears to inconveniently promote atopic dermatitis in children [12,13]. According to the most recent reviews of published data, VD3 supplementation during childhood reduces the risk of the exacerbation of asthma, atopic dermatitis, and rhinitis symptoms. Hence, the role of the endogenous level of VD has been envisaged for the first time as a critical factor in allergies [14]. However, a recent clinical trial (randomized and controlled) did not support the use of VD3 supplementation to prevent exacerbations in a group of children with low VD levels affected by severe asthma [15]. Conversely, in non-atopic toddlers, VD3 supplementation was associated with a higher risk of developing asthma [17], allergic rhinitis, and atopic dermatitis (Table 1).

Nevertheless, we described an adjuvant role of VD3 in Der p 2-based experimental MA-AIT in a mouse allergy model [20]. More recently, an observational study conducted by us showed that one-year AIT, besides resulting in the expected amelioration of clinical scores, also increased memory effector Tregs [10]. Furthermore, in asthmatic children undergoing AIT, a combination treatment with VD3 reduced rhinitis and asthma [16] and further enhanced the increase in Tregs, IL-10, and TGF-β [18,19]. In addition, a few trials by other investigators have shown inspiring, although not robust, results that foresee the clinical use of VD3 in allergy management [19].

Hence, we sought out potential roles of VD in the severity of allergies, in the clinical outcomes of effective AIT, and in the underlying immunomodulatory mechanism by conducting post hoc analyses on three different cohorts of mite-allergic children.

**Figure 3:**
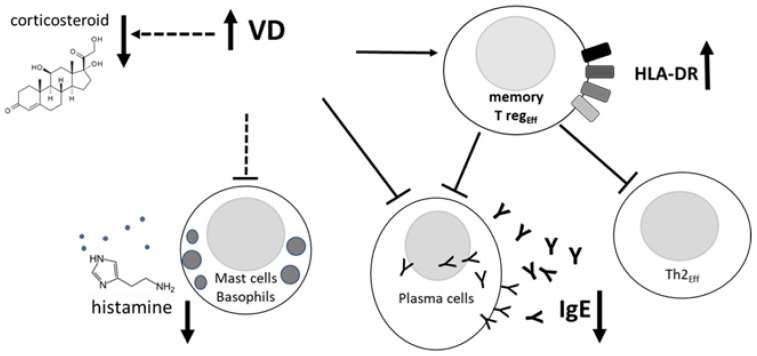
Hypothetical VD role in the context of effective AIT in low-VD children.

Our post hoc analyses unveiled the result that AIT is more effective in children with a fully sufficient serum VD level (>27 ng/mL), either naturally occurring or achieved by supplementation, as shown by the uppermost amelioration of the allergic symptoms of rhinitis and asthma, the lowest corticosteroid requirement, and the diminution in specific IgE levels (Table 2). Furthermore, VD might provide empowerment to the inhibitory function of effector memory Tregs through the hypothetical mechanism described in Figure 3. A few takeaway messages can be drawn from our findings: endogenous serum VD levels appear to be inversely associated with allergy severity and medication consumption; furthermore, a sufficient endogenous VD level is associated with better improvements in AIT efficacy. Additionally, VD might play an indirect role in the AIT context by conditioning the activity of the immune cells involved in the underlying immunological mechanism (Figure 3).

A recent study provided supporting data for our point of view, since it showed that a better built-up efficacy of allergy immunotherapy was achieved in VD3-supplemented allergic patients [17]. Clinical studies have indicated that serum VD levels are inversely associated with the responsiveness to corticosteroids, with a lower VD level corresponding with a higher drug consumption [24]. Furthermore, dexamethasone-resistant patients showed IL-10-unproductive Tregs [25]. In addition, IL-10 production and the sensitivity to glucocorticoids were restored upon the co-administration of VD3 in those patients [25]. VD3 also induced Tregs in vitro, displayed an additive effect with glucocorticoids, and was associated with the reversal of glucocorticoid resistance [26]. A randomized clinical trial excluded VD3 as an adjuvant supplement for (non-allergic) asthma management, which did not disprove our results since the trial was conducted in non-allergic adults with a VD insufficiency [27].

## 6. Conclusions

We conclude that the clinical outcomes of AIT are not affected by endogenous levels of VD and VD3 supplementation. Nevertheless, our findings suggest that it might be useful as an add-on supplement in children with VD deficiency/insufficiency undergoing AIT and characterized by a high need for symptomatic medications. VD’s role in maintaining antigen-specific, long-term, Treg-mediated tolerance to an eliciting allergen is worthwhile for evaluation in the unexplored setting of AIT clinical trials.

Some limitations of our post hoc analysis are the single-center geographical area /population and the modest cohort size. Despite that, our investigation deals with a hot and interesting topic and may provide hints for future clinical trials. Further experimental multicentric clinical trials are envisaged to better address the role of endogenous and exogenous VD. Serum VD levels and peripheral Treg immunophenotyping could be valuable markers for monitoring AIT during treatment. We also highlight the idea that the immunogenic/low-allergenic mite monomeric carbamylated allergoid used as a bioactive agent might have contributed to minimizing the allergic responses and highlighting the immunological effects described here.

## Figures and Tables

**Table 1:** Exogenous VD, as a food or food supplement, on allergic and respiratory diseases in children, according to clinical trials and meta-analyses.

	Type of Study	Observation of VD	Atopic Dermatitis	Allergic Rhinitis	Allergic Asthma	Non-Atopic Asthma	References
**Atopic** **Children**	Large-cohort epidemiological studies	VD-rich food (mothers during pregnancy)	Promotion	Prevention of incidence	Prevention of incidence	-	[12,13]
**Atopic Children**	Systematic review meta-analysis	VD3 supplementation (and endogenous)	Reduction in the risk	Reduction in the risk	Reduction in the risk	-	[14]
**Atopic Children** **(Low Endogenous VD)**	Randomized controlled clinical trial	VD3 supplementation	-	-	No prevention of severe asthma exacerbations	-	[15]
**Allergic** **Children** **on AIT**	Randomized controlled clinical trial	VD3 supplementation	-	Reduced	Reduced	-	[16]
**Non-Atopic** **Children**	Nested case-control study	VD3 supplementation	Higher risk of development	Higher risk of development	Higher risk of development	-	[17]

**Table 2:** Anthropometric and clinical parameters of allergic patients.

Gender (female, F; male, M)	80 F, 85 M
Age (years)	10.4 ± 3.1
Weight (kg)	42.7 ± 15.5
Height (cm)	144 ± 18.0
Body mass index (w/h^2^) (kg/m^2^)	19.4 ± 4.3
Weight at birth (kg)	3.2 ± 0.5
Breastfeeding (months)	7.5 ± 5.4
Age at weaning (months)	5.0 ± 0.8
Gestational age (weeks)	39.3 ± 1.7
Neonatal respiratory distress n. (%)	7 (5)
Parental history of allergy/asthma n. (%)	79 (61)
Symptomatic allergic patients n. (%)	165 (100)

Data are presented as absolute values, % of total patients, or mean + standard deviations, as indicated.

**Table 3:** Serum VD level, allergy, and respiratory data of the series of patients (N = 70) were included in post hoc analysis 1 (allergic, no AIT).

Serum	
VD (ng/mL)	22.0 ± 5.0
Mite-specific immunoglobulin E	
Df IgE (kUA/L)	48.5 ± 35.0
Dp IgE (kUA/L)	59.3 ± 33.6
Respiratory scores	
ARIA	3.3 ± 0.6
ACT	20.6 ± 2.4
VAS	7.6 ± 1.5
Medications	
Oral antihistamines Yes/No (%)	70/0 (100)
ICS-LABA Yes/No (%)	44/26 (63)

Data are presented as absolute values, % of total patients, or mean ± S.D., as indicated.

**Table 4:** Allergen specificity and ICS-LABA usage of the two sub-cohorts of patients, grouped according to the mean serum VD level (22 ng/mL used as the cut-off value) at the time of diagnosis.

Serum VD Level (ng/mL)	<22	≥22	
No. of patients	N = 38	N = 32	*p*
Df IgE (kUA/L)	63.6 ± 30.5	34.7 ± 22.8	<0.0001
Dp IgE (kUA/L)	65.1 ± 32.1	42.5 ± 33.0	=0.0051
ICS-LABA Yes/No (%)	32/6 (84.0)	12/20 (37.5)	<0.0001

Only significant differences (*p* < 0.005) are shown. Data are presented as absolute values, % of total patients, or mean + standard deviations, as indicated.

**Table 5:** Serum VD level, allergy, and respiratory data of the AIT group of patients (N = 60) at diagnosis and after 12 months of treatment.

	Pre-AIT	Post-AIT
VD (ng/mL)	21.8 ± 4.7	27.0 ± 5.1
Df IgE (kUA/L)	49.4 ± 33.0	45.9 ± 33.5
Dp IgE (kUA/L)	60.2 ± 34.1	56.2 ± 34.2
Oral antihistamines Yes/No (%)	60/0 (100)	5/55 (8.3)
ICS-LABA Yes/No (%)	38/22 (64)	3/57 (5)

**Table 6:** Levels of specific IgE values in response to Der f and Der p for the two sub-cohorts of AIT patients (N = 60) according to the cut-off VD level (27 ng/mL), 12 months post-treatment.

Serum VD Level (ng/mL) (Post-AIT)	<27	≥27	*p*
No. of patients	(N = 28)	(N = 32)	
Der p IgE (kUA/L)	55.3 ± 32.6	56.9 ± 36.4	n.s.
Der f IgE (kUA/L)	52.4 ± 31.6	40.9 ± 36.0	n.s.
VAS	3.2 ± 2.2	1.7 ± 1.2	<0.0001
Oral antihistamines Yes/No (%)	6/22 (21)	0/32 (0)	<0.0001
ICS-LABA Yes/No (%)	2/26 (7.0)	2/30 (6.0)	n.s.

**Table 7:** Absolute values and statistical significance of time-dependent and VD3-dependent changes in relevant clinical parameters of the two study groups (+VD3 and −VD3) of post hoc analysis 3.

	+VD3 (on AIT)			−VD3 (On AIT)			VD3-Dependent Significance (Post-AIT)
Parameter	Baseline (N = 19)	12 Months (N = 16)	*p*	Baseline (N = 16)	12 Months (N = 14)	*p*	*p*
25-OH VD (ng/mL)	20.1 ± 4.2	36.1 ± 2.8	#	22.4 ± 4.2	23.2 ± 3.0	n.s.	§
ARIA	3.5 ± 0.6	0.9 ± 0.4	#	3.5 ± 0.6	1.5 ± 1.0	#	§
VAS	8.2 ± 1.1	1.7 ± 1.2	#	7.5 ± 1.7	3.2 ± 2.2	#	§
ACT	20.7 ± 3.8	25.1 ± 0.7	#	21.1 ± 4.6	25.2 ± 1.1	#	n.s.
Oral antihistamines Yes/No (%)	19/0 (100)	0/16 (0)	#	16/0 (100)	3/11 (21.4)	#	§
ICS-LABA Yes/No (%)	16/3 (84)	1/15 (6.2)	#	6/10 (37.5)	1/13 (7.1)	#	§
Nasal corticosteroids Yes/No (%)	6/13 (31.6)	0/16 (0)	#	6/10 (37.5)	1/13 (7.1)	#	n.s
Antileukotrienes Yes/No (%)	4/15 (21)	0/16 (0)	#	3/13 (18.7)	0/14 (0)	#	n.s

Data are expressed as the number of patients who showed improvement in the indicated clinical score divided by the total number of patients ± S.D. #: *p* < 0.05 (baseline vs. 12 months); §: *p* < 0.05 (+VD3 vs. −VD3); and n.s.: not significant. *p* values were calculated using the non-parametric Wilcoxon signed rank test.

**Table 8:** Significance of delta changes (baseline–12 months) of the clinical and immunological parameters, and the medications needed, according to VD3 supplementation.

	Tendency of Clinical Improvement		Significance of the Differences between ±VD3
	−VD3 (N = 16)	+VD3 (N = 19)	*p*
**ARIA**	−2.0 ± 1.0	−2.5 ± 0.7	*p* = 0.0272
**VAS**	−4.4 ± 1.2	−6.3 ± 1.5	*p* < 0.0001
**Oral antihistamine**	−0.78 ± 0.42	−1.0 ± 0.1	*p* = 0.0056
**ICS-LABA**	−0.2 ± 0.4	−0.7 ± 0.4	*p* < 0.0001

**Table 9:** Comparison between baseline values and respective post-AIT values of IgE serum levels in the two sub-cohorts, +VD3 and −VD3.

	+VD3 (on AIT)			−VD3 (on AIT)		
Parameter	Baseline (N = 19)	12 Months (N = 16)	*p*	Baseline (N = 16)	12 Months (N = 14)	*p*
Total IgE (kU/L)	692.3 ± 723.2	444.9 ± 366.8	#	492.0 ± 529.4	385.0 ± 366.2	n.s.
Dp IgE (kUA/L)	60.0 ± 32.1	56.9 ± 36.4	n.s	44.7 ± 29.0	55.3 ± 32.6	n.s.
Df IgE (kUA/L)	61.1 ± 32.9	40.9 ± 36.0	#	35.4 ± 25.4	52.4 ± 31.6	n.s.

#: *p* < 0.05 (baseline vs. 12 months).

**Table 10:** Recapitulation of the findings, according to the sets of data analyzed.

Post Hoc Analysis 1 Allergic			Post Hoc Analysis 2 AIT Treatment		Post Hoc Analysis 3 AIT Treatment ±VD3		
Endogenous VD	Symptoms	Medications	Endogenous VD	Primary End-Points	Supplemented or Endogenous VD	Primary Clinical End-Points	Surrogate End-Points (Memory Treg)
<22 ng/mL	More severe	Higher ICS-LABA	<27 ng/mL	Optimal clinical scores except for OA and VAS	−VD3 or not sufficient	Optimal scores	Lower HLA on memory Tregs
≥22 ng/mL	Less severe	Lower ICS-LABA	≥27 ng/mL	Optimal clinical scores	+VD3 or sufficient (>30 ng/mL)	Optimal scores	Higher HLA on memory Tregs

## Data Availability

Not applicable.
